# Paradoxes of Difference

**DOI:** 10.1371/journal.pbio.0020263

**Published:** 2004-09-14

**Authors:** Sandra Soo-Jin Lee

## Abstract

A new play tackles the politics around race and gender in science

[Fig pbio-0020263-g001]

**Figure pbio-0020263-g001:**
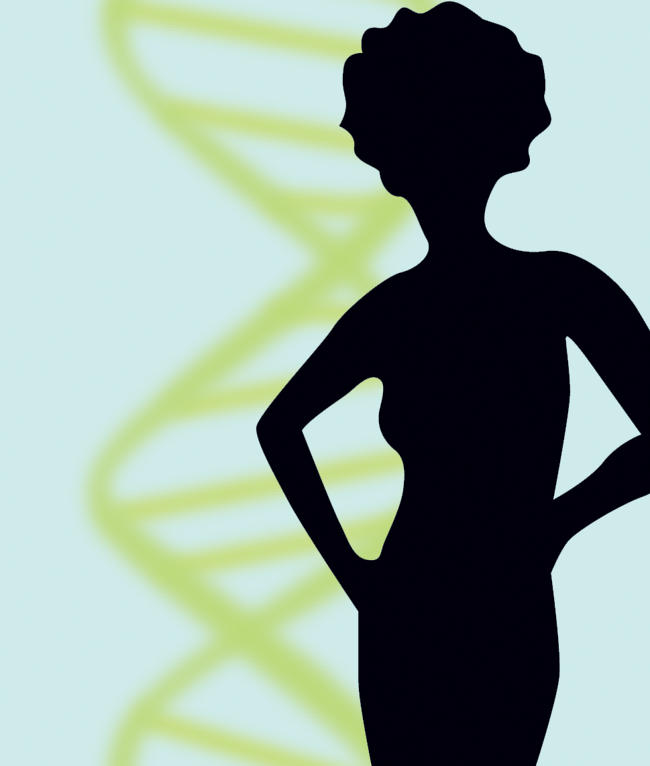


Can “race” be gleaned from our genes? Concern over the emerging trajectories of genetics research has led to ongoing debates about how to characterize and interpret genetic variation. Despite the mantra that humans share 99.9% of our genetic makeup, there is increasing interest in identifying the relatively small percentage difference that distinguishes individuals. Therein lies the paradox: if we are all the same, why do we continue to search for the ways in which we differ from one another? We can take an essential first step toward addressing this paradox by acknowledging the often conflicting stakes for individuals and groups in debates that center around genes, science, and race. Whose genes will be studied? For what purpose? And who has the authority to decide?

These stakes are laid bare by Cassandra Medley in her play “Relativity,” which ran at the Magic Theater in San Francisco this spring, in a production directed by Edris Cooper-Anifowoshe. The play focuses on the inner conflict of a young scientist, an African American woman who tries unsuccessfully to straddle the opposing world views of her profession and her family. In “Relativity,” Kalima Davis is a postdoctoral fellow in a prestigious stem cell laboratory on the East Coast. Her impeccable academic pedigree distinguishes Kalima as a rising researcher, and she is one of the few women and African American scientists working in the field. Kalima is also the devoted daughter of the charismatic psychotherapist Claire Reid, who directs the “Leon Davis Foundation,” which Kalima's late father dedicated to the belief that neuropeptide melanin, found in “people of color,” enhances intelligence, athleticism, and emotional sensitivity. This theory also points to the lack of melanin among lighter skinned individuals as a cause of “white racism.” Kalima, who has inherited co-directorship of her father's foundation, is asked by her mother to offer “scientific proof” of the melanin theories and to discount the assertion that all groups are genetically similar.

At issue in “Relativity” is the struggle over what constitutes a valid belief. Reid suggests to Kalima that “science is not the sole province of what the ‘West’ defines it to be,” and refers to Chinese acupuncture, Hindu Chakra, and tribal African shamanism as examples of legitimate “sciences.” But the power of Western science to trump other interpretations of lived experience has become all too clear in the genomic era. Truth is excavated from the human body, where genes emerge as the iconographic oracles of our past, present, and future. As Kalima recounts, “We can't get around it. DNA is fact.” Nonetheless, Kalima naively attempts to find a way to retain these opposing epistemologies. The futility of this dual position is made apparent by the arrival of Iris Preston, an African American senior scientist who has taken over as the new head of Kalima's lab. Preston, a highly vocal critic of Claire Reid and melanin theories, serves as a formidable apostle of the scientific method. Shortly after her arrival, Preston convenes members of her new lab to filter those seeking financial and other derivative rewards from the truly devoted, who are motivated solely by their “sense of wonder and amazement” and their desire to “cultivate what Einstein referred to as ‘holy curiosity.’” She makes plain that science, like all ideologies, demands consummate faith and unwavering piety.

Kalima's struggle to claim her “true lineage” is much more than a simple choice between her biological mother (Reid) and her intellectual mentor (Preston). Her plight forces her to explore the meaning of justice. The contrast between melanin theories and genomic research, initially stark, blurs as it becomes increasingly clear that both Reid and Preston seek to use their “science” to redress race-related disparities. Citing a history of racism, including the historically well-documented Tuskegee Syphilis Study, Reid asks rhetorically whether genetics research will result in a “genetically modified” white upper class and a lower, dark-skinned “natural birth class”? At stake are issues of power and trust, and the question of whether new genetic technologies will close the gaps between groups or make them wider. Some postulate that the “new genetics” will render conventional notions of race obsolete. However, it is doubtful that such a color-blind utopia will be won through the sequencing of genes without a serious engagement with the differences that lie outside cell walls. A social infrastructure of inequality that mediates race through nutritional deficits, exposure to pollutants, and the use of the emergency room as the sole venue for healthcare will not be rehabilitated through gene therapy or pharmacogenomics.

Preston seeks to use her stature in science to focus on the inequities within the academy. Encouraging Kalima to appear with her on a television program about stem-cell research, Preston urges her to imagine the milestone of “not just one, but two black women scientists, holding forth among the usual cadre of white males.” As one of the rare, highly scrutinized, “minority” scientists, Kalima embodies a “double jeopardy.” She must prove that she is worthy of her position—that she is as good, if not better, than her “white” peers—yet she must always be “remembering from whence she came.” When Dan, a white colleague in Kalima's lab who is also her boyfriend, hears that Preston has chosen Kalima to appear with her on television, he jealously accuses her of benefiting from preferential treatment. The play's depiction of this assertion of “reverse racism” questions the legitimate use of race in evaluating promotion and achievement in science. What is apparent is that race, while an ever-present subject, is often presented as the antithesis of conventional notions of a color-blind meritocracy. Left to linger is the critical question: does merely identifying differences among groups constitute an act of racism? Can statements of racial differences be neutral, unfettered by a relative hierarchy?

Medley throws into stark relief the politics around race and gender in science. She illuminates how an individual's dilemma transcends the private realm; personal decisions are never truly “personal,” but are inherently public because they always have wider social repercussions. In the fateful confrontation between Kalima and her mother, Reid challenges her daughter to “grow up” and to risk her disapproval. A similar challenge can be issued to those who have inherited the “new genetics”—to vanquish the continuing paradox of reciting a “mantra of sameness” all the while searching for meaningful differences. In “Relativity,” Medley serves us a cautionary tale of the costs of our chronic ambivalence about the critical issues of race and justice in science. A good first step is to recognize that the search for meaning in human difference is inseparable from the struggle over the moral order in which we live.

To underestimate the power of science to define our social agenda is to lose an opportunity to determine our future course. We only need to look to history as our proof.

